# Gene expression profiling and construction of a putative gene regulatory network of bladder cancer tumor-initiating cells

**DOI:** 10.18632/oncotarget.22771

**Published:** 2017-11-30

**Authors:** Zhuoyuan Xin, Zhao Yang, Jianting Xu, Chaoying Li, Tong Shao, Guoqing Wang, Chong Li

**Affiliations:** ^1^ Cancer Centre, First Hospital of Jilin University, Changchun, China; ^2^ Department of Pathogenobiology, College of Basic Medical Science, Jilin University, Changchun, China; ^3^ Core Facility for Protein Research, Institute of Biophysics, Chinese Academy of Sciences, Beijing, China; ^4^ Beijing Jianlan Institute of Medicine, Beijing, China

**Keywords:** bladder cancer, gene expression profile, tumor-initiating cells, stem-like cells, gene regulatory network

## Abstract

Human bladder cancer tumors have been shown to contain a subpopulation of cells with stem-like characteristics that may trigger tumor growth, recurrence, and metastasis. These cells, known as tumor-initiating cells (TICs), would be effective diagnostic tools and valuable therapeutic targets. Here, we report the isolation of TICs from seven bladder cancer cell lines and show that TICs from different sources vary on their ability to form tumorspheres *in vitro* and generate xenografts *in vivo*, which suggest they are remarkably heterogeneous. We used the Affymetrix PrimeView™ Human Gene Expression Array to analyze gene expression profiles of bladder TICs, which may help understand their tumorigenic capacities and develop novel treatments specifically targeted toward these cells. We then constructed a transcription factor-gene regulatory network that includes three key transcription factors that are involved in cell survival, differentiation, proliferation, and apoptosis. We validated our findings by analyzing mRNA expression of the key genes in this network in 24 clinical tissues. Our results suggest that this transcription factor-gene regulatory network could be useful in the development of clinical diagnostic tools and therapy approaches for bladder cancer.

## INTRODUCTION

Bladder cancer is the second most prevalent malignancy of the urinary system. Globally, an estimated 429,793 new cases were diagnosed and 165,068 deaths from bladder cancer occurred in 2012 [1-4]. About 50% to 70% of bladder cancer patients who undergo a radical surgical procedure may experience recurrence and overall deterioration and lead to distant metastases within 5 years [5]. Presently, the specific mechanisms of bladder cancer growth, invasion, and metastasis are unclear; therefore, effective early diagnosis tools and treatments are lacking. Increasing evidence has suggested that a heterogeneous subpopulation of cells with stem-like characteristics, termed tumor-initiating cells (TICs), is responsible for maintaining tumor growth and possess strong capacity of self-renewal and invasion, which result in recurrence and metastasis [6-10]. Hence, functional characterization of such tumor-initiating cells may provide valuable information to be used in clinical settings [11]. The use of cell surface markers for sorting bladder TICs is still controversial. CD44 is a transmembrane glycoprotein with a highly conserved N-terminal domain involved in several vital cellular processes, including cell proliferation, survival, differentiation, and motility. In response to the extracellular microenvironment, CD44 is a unique adhesion molecule that plays an essential role in cancer cell migration and matrix adhesion. CD44-positive cells have been suggested to function as cancer TICs in bladder cancer tissues and bladder cancer cell line T24, which possesses high tumorigenicity and metastatic potential [12, 13]. However, specific molecular markers may express differentially in different bladder TICs, bladder cancer cell lines isolated from diverse bladder cancer subtype tissues are highly heterogeneous [14]. In addition, tumor formation capacity and the signal pathways involved in the regulation of stem-like properties may vary considerably in different bladder cancer subtypes. In a number of bladder TICs, continuous growth and self-renewal are closely related to aberrant gene expression and altered gene regulatory networks [15]. Therefore, high-throughput screenings to identify differential gene expression profiles and signaling regulatory networks in different bladder TICs, and the analysis of TICs heterogeneity and their specific central regulatory molecules should provide valuable information for the development of individualized target therapies for bladder cancer.

In this study, we have sorted TICs from seven bladder cancer cell lines and analyzed their heterogeneity. Using the Affymetrix PrimeView™ Human Gene Expression Array, we identified the gene expression profiles of different bladder TICs. We further constructed a transcription factor-gene transcription regulatory network to analyze the similarities and differences in gene expression and signaling regulatory pathways among different bladder TICs. Our data will be invaluable in future studies of carcinogenic processes and regulatory mechanisms of bladder TICs and lay de foundation for the identification of novel clinical therapeutic targets.

## RESULTS

### Isolation and identification of bladder TICs

#### Isolation of a subpopulation of cells with stem-like characteristics from bladder cancer cell lines

**Figure 1:**
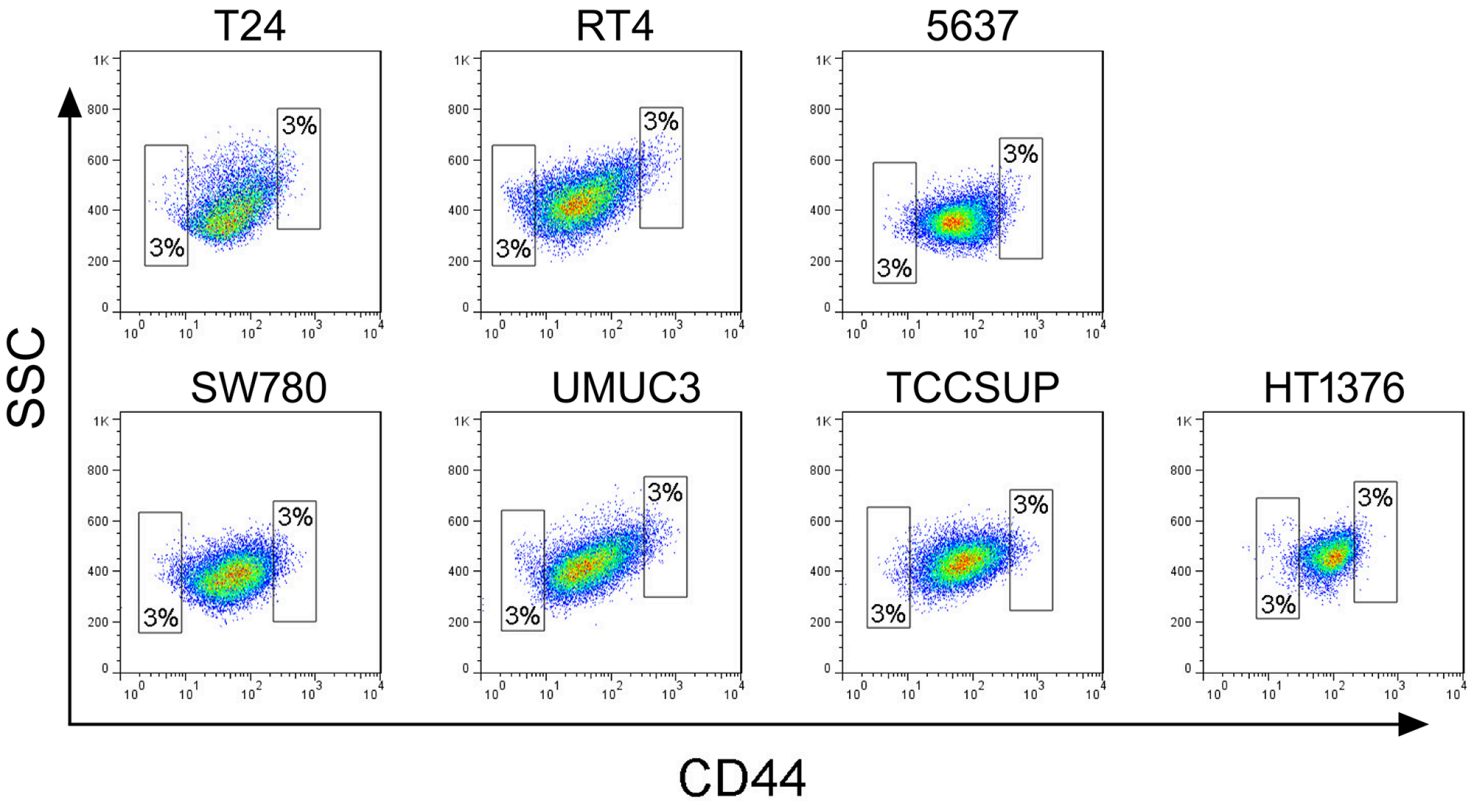
FACS of CD44-positive cells in seven bladder cancer cell lines CD44^high^ and CD44^low^ cell subpopulations were isolated from seven cell lines using an anti-CD44 antibody. In each bladder cell line, CD44^high^ and CD44^low^ subpopulations account, each, for 3% of the total number of cells.

**Figure 2:**
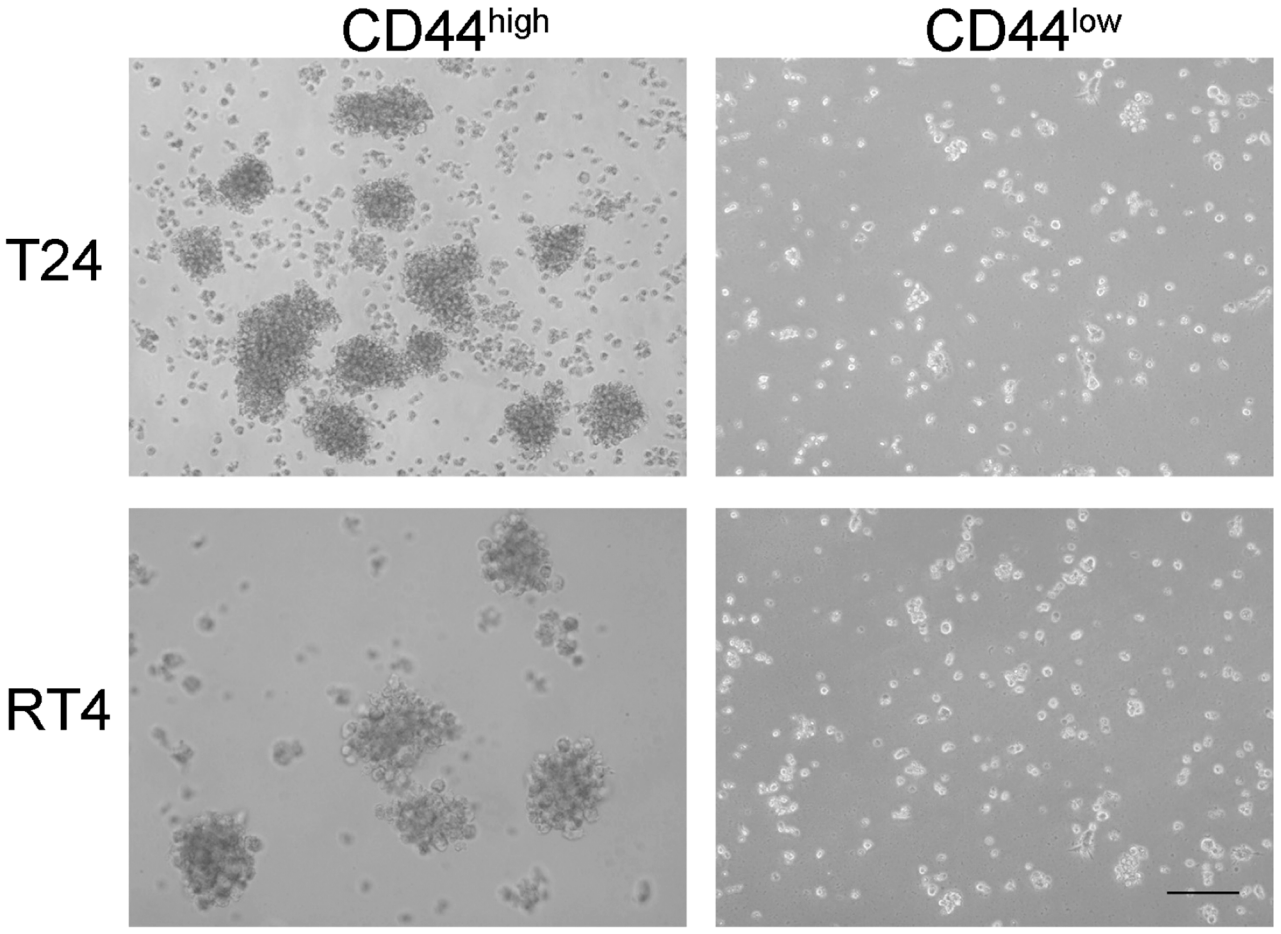
Cancer stem cell sphere formation assay on CD44high and CD44low cells from two different bladder cancer cell lines CD44^high^ cells formed oncospheres (left panels) while CD44low cells did not form oncospheres (right panels) when cultured in medium for the same period of time.

To determine whether tumor-initiating cell subpopulations existed in different bladder cancer cell lines, we first isolated CD44-positive cells from seven human bladder cancer cell lines, including T24, 5637, RT4, SW780, UM-UC-3, TCCSUP, and HT-1376 (Supplementary Table 1). As shown in previous studies, cells within the topmost 3% of CD44-positive signal intensity were defined as the “CD44^high^” subpopulation, and were preliminarily identified as bladder TICs on the basis of their high sphere formation capacity. Cells within the lowest 3% CD44-positive signal intensity were identified as the “CD44^low^” group. CD44^high^ and CD44^low^ subpopulations were sorted (Figure 1) and separately cultivated in medium. All CD44^high^ subpopulations sorted from different bladder cancer cell lines were able to form oncospheres (Figure 2); however, they varied in their ability to proliferate and form colonies.

#### CD44^high^ cells show increased tumorigenicity

**Figure 3:**
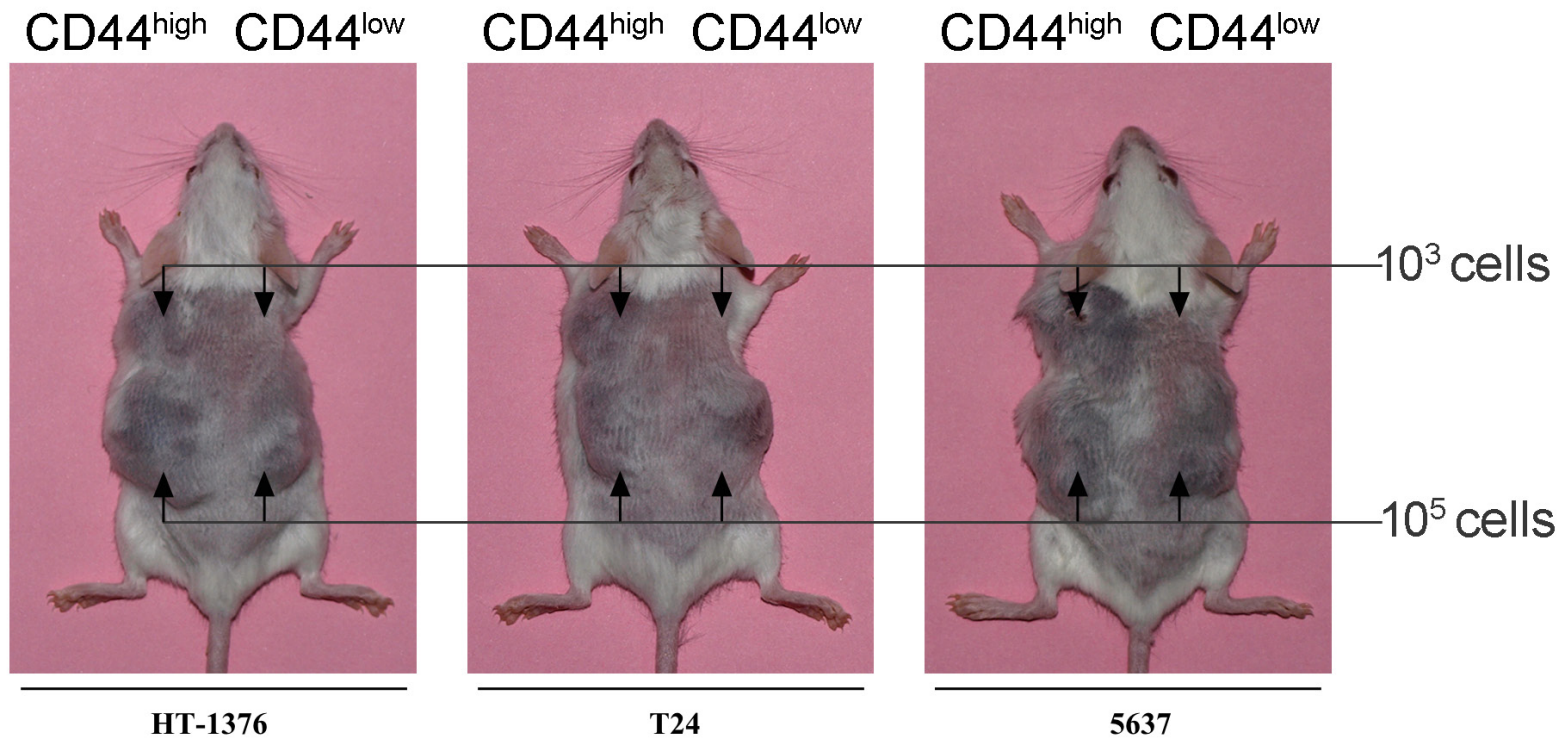
Generation of CD44^high^ and CD44^low^ xenografts in NOD/SCID mice Each mouse was intradermally injected with 10^3^ (upper body) and 10^5^ (lower body) CD44^high^ (left side) andCD44^low^ (right side) cells obtained from one of the seven bladder cell lines. CD44^high^ cells (TICs) from different bladder cell lines showed heterogeneous capacity of xenograft generation *in vivo*.

To further identify putative bladder TICs, reliable *in vivo* xenograft models were established. Suspensions of freshly isolated bladder CD44^high^ and CD44^low^ cells were intradermally injected into non-obese diabetic/severe combined immunodeficiency (NOD/SCID) mice, which lack T, B, and NK cells. Simultaneously, unsorted cell suspensions were also injected as control. CD44^high^ cells from all seven bladder cancer cell lines successfully generated xenograft tumors (success rate: 20 to 70%) with an average latency of a month. In contrast, CD44^low^ cells and unsorted cells did not form transplanted tumors (Figure 3 and Table 1). We found that the engraftment capacity of CD44^high^ cells was clearly heterogeneous. Seventy percent of the mice injected with CD44^high^ cells from the T24 cell line generated xenografts; in contrast, only 20% of the mice injected with CD44^high^ cells from the HT376 cell line formed transplanted tumors. Engraftment ability could be associated with tumor invasion, distant metastasis and age of patients (i.e., the more invasive the original tumor and the older the patient, the more likely that xenograft tumors will be generated). It is possible that advance stage tumors and a deteriorated microenvironment induces stem-like and invasive characteristics in tumor cells for survival.

**Table 1:** Number of xenografts generated in NOD/SCID mice after injection of CD44^high^ and CD44^low^ cells isolated from different bladder cancer cell lines

Type of cells	Bladder cancer cell line						
	T24	RT4	5637	SW780	UMUC3	HT1376	TSSCUP
**Unsorted**	0/10	0/10	0/10	0/10	0/10	0/10	0/10
**CD44^high^**	7/10	5/10	3/10	3/10	5/10	2/10	5/10
**CD44^low^**	0/10	0/10	0/10	0/10	0/10	0/10	0/10

A total of 10^3^cells/mouse were injected intradermally. Mice were monitored for at least 5 months before being considered as negative (0) for engraftment.

Ten of xenografts/number of injected mice is indicated.

### Gene expression profile analysis of bladder TICs

#### Identification and functional annotation of differentially expressed genes

**Figure 4:**
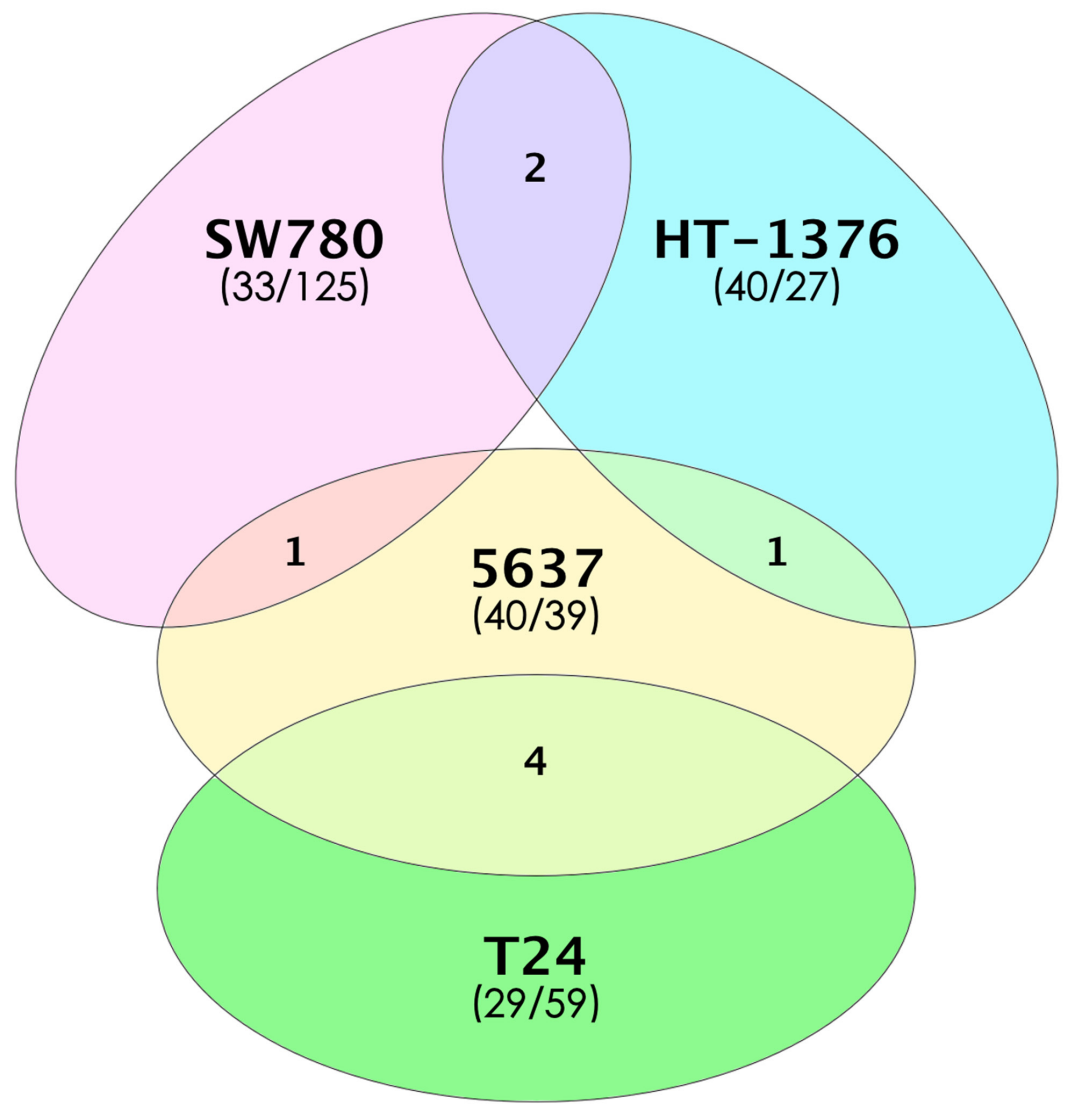
Number of differentially expressed genes in four bladder TICs Overlapping regions between ovals indicate the number of differentially expressed genes shared by two bladder TICs. The number of up-regulated/down-regulated genes for each bladder TIC is indicated in parentheses.

To characterize bladder TICs at the molecular level, we used the Affymetrix PrimeView™ Human Gene Expression Array to analyze four bladder cancer cell lines (SW780, HT-1376, T24, and 5637). Upon removal of duplicate genes, 216,265 genes remained for further analysis. We identified 67, 79, 88, and 158 genes showing a >2-fold change in expression in bladder TICs isolated from SW780, HT-1376, T24, and 5637, respectively (*p*<0.05; Supplementary Table 2). Surprisingly, none of the differentially expressed genes was common to all four bladder TICs; however, a limited number of differentially expressed genes were shared between two different bladder TICs (Figure 4 and Supplementary Table 3). This observation supports the idea that different bladder TICs are heterogeneous.

#### Construction of a bladder TICs-related transcription factor-gene regulatory network

**Figure 5:**
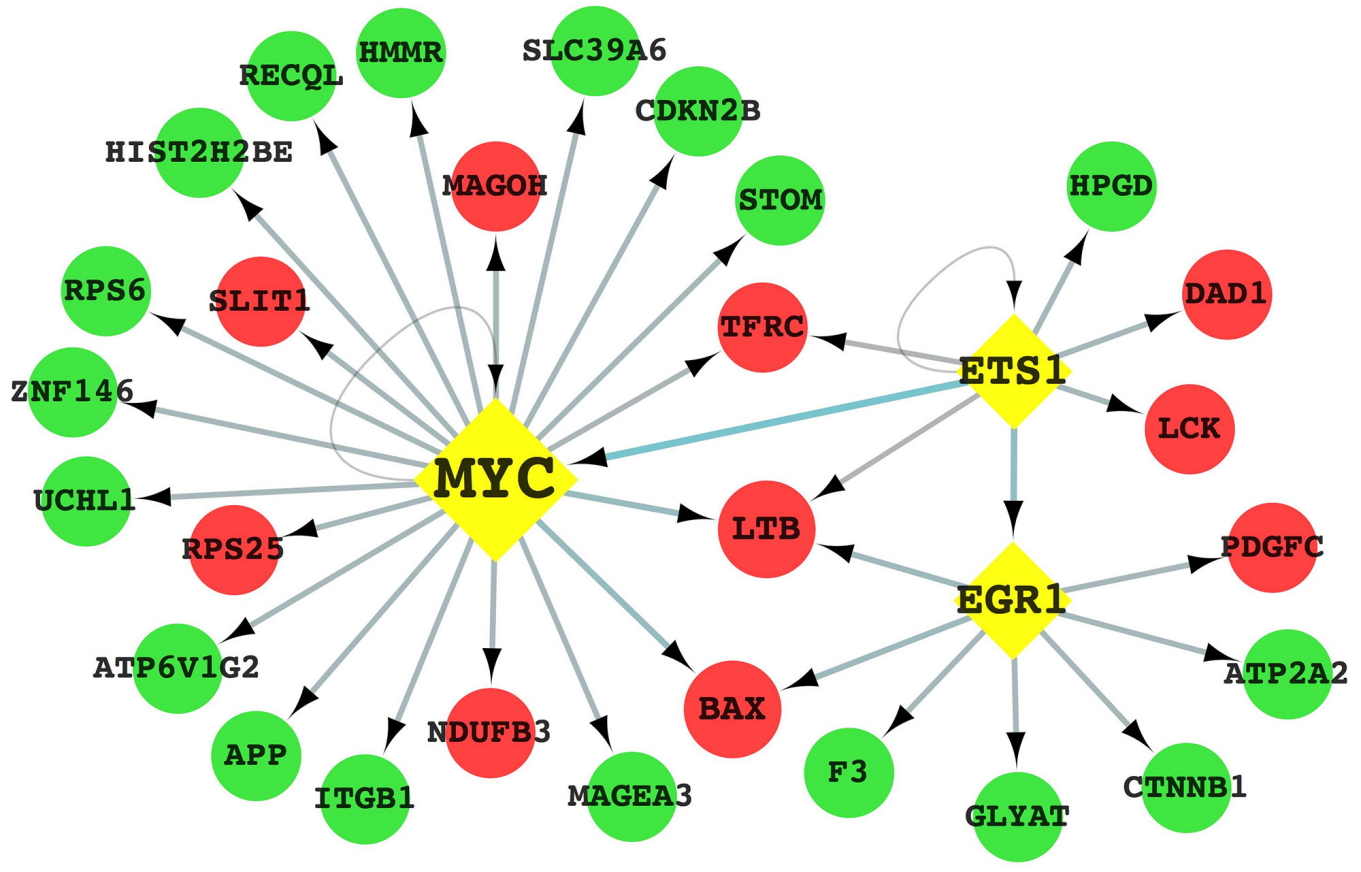
Transcription factor-gene regulatory network potentially involved in the regulation of bladder TICs characteristics Circles in red indicate up-regulated genes, whereas green circles indicate down-regulated genes. The yellow diamonds represent the three key transcription factors. Arrows represent interactions between TFs and genes.

The interaction between transcription factors (TF) and genes is an essential step in gene expression regulation. TF-gene interactions may be significantly altered in anomalous tissues. Therefore, in order to define a universal set of diagnostic targets for bladder cancer, we combined all the differently expressed genes identified in the four bladder TICs with data from TRED (https://cb.utdallas.edu/cgi-bin/TRED/tred.cgi?process=home) [16] and constructed a transcriptional regulatory network (Figure 5, Supplementary Table 4). Utilizing The Database for Annotation Visualization and Integrated Discovery (DAVID) [17, 18], genes involved in the regulatory network were annotated with Gene Ontology (GO) items. And then KEGG pathway enrichment analysis was performed to analyze how this transcription factor-gene regulatory network driven the generation of bladder TICs (Supplementary Figure 1).

### Validation of bladder TICs transcription factor-gene regulatory network in bladder cancer tissues

**Figure 6:**
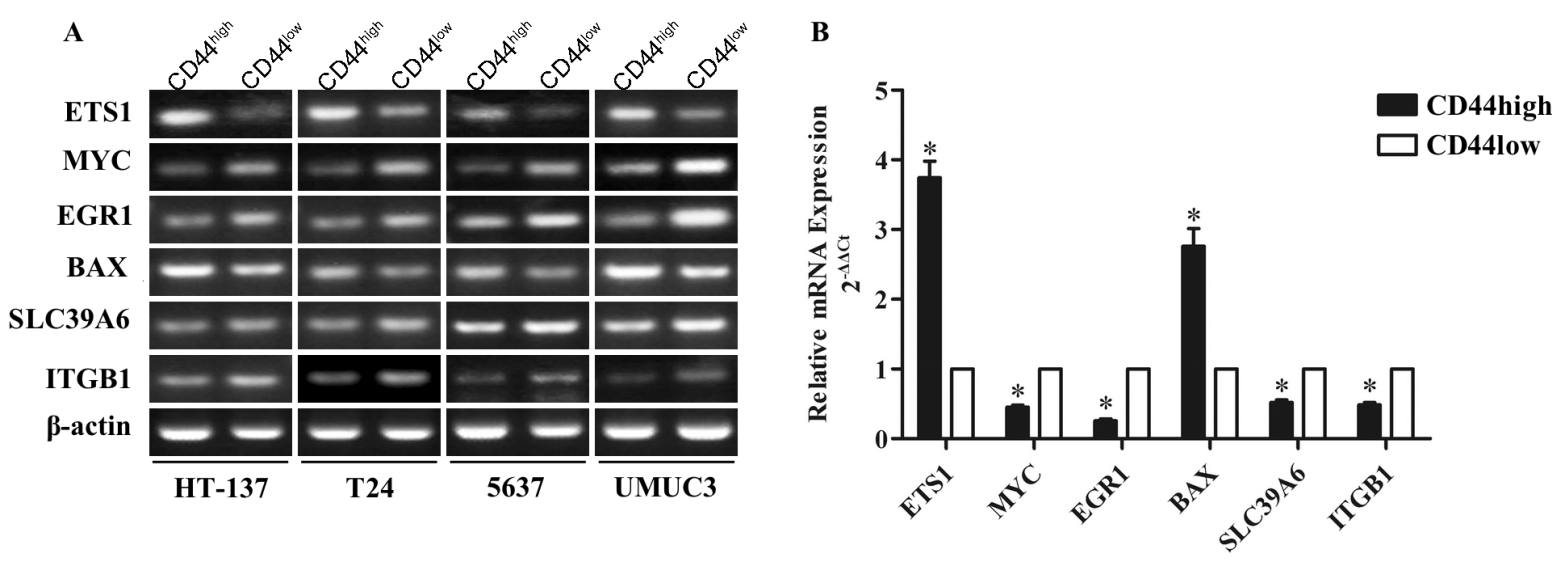
mRNA expression analysis of six genes from the bladder TICs TF-gene regulatory network in 24 pairs of bladder cancer CD44^high^ *vs.* CD44^low^ tissues *ETS1*, *MYC*, *EGR1*, *BAX*, *SLC29A6*, and *ITGB1* mRNA expression was analyzed in bladder cancer CD44^high^ *vs.* CD44^low^ tissues using PCR **(A)** and qRT-PCR **(B)**. ^*^p<0.01.

The TF-gene regulatory network constructed in this study was based on gene expression profile data obtained from bladder cancer cell lines. Therefore, to validate the candidate targets in the network in bladder cancer tissues, we sorted tissue cells based on their level of expression of the CD44 marker and performed qRT-PCR to examine the level of mRNA expression of three genes (*BAX*, *SLC39A6*, and *ITGB1*), as well as the three key TFs (*ETS1*, *MYC*, and *EGR1*) in the network. We analyzed 24 bladder cancer tissue sample pairs and found that *ETS1* and *BAX* were up-regulated (3.7417±1.1395, 2.7592±1.2276 Fold respectively, P<0.01), whereas *MYC*, *EGR1*, *SLC39A6*, and *ITGB1* were down-regulated (2.2163±0.1357, 3.8834±0.1242, 1.9277±0.1792 and 2.0565±0.1459 Fold respectively, P<0.01) in bladder cancer CD44^high^ *vs.* CD44^low^ tissue cells (Figure 6). These results validate our TF-gene regulatory network, suggesting that the network constructed in this study regulates the general characteristics of bladder TICs. Hence, genes identified in this network may be useful novel potential clinical therapeutic targets and prognostic markers for bladder cancer.

## DISCUSSION

In this work, we confirmed that the sphere formation ability of the CD44^high^ subpopulation might be positively correlated with distant metastasis of original patient specimens and tumor invasion. Our findings suggest that high expression level of the cell surface protein CD44 is a useful biomarker of bladder TICs. We systematically analyzed the differentially expressed genes from bladder TICs by gene ontology enrichment analysis to functionally annotate and predict their roles [19]. We performed the functional analysis for clustering the genes with related biological functions [18]. Up-regulated genes in bladder TICs were correlated with diverse biological processes, such as macromolecular complex assembly, response to toxins, regulation of organelle organization, and nucleosome assembly. Moreover, 15 up-regulated genes were associated with extracellular space, including *ADAMTS1*4, *C1QTNF7*, *APOB*, and *EPGN*, and might be involved in extracellular matrix remodeling (See Table 2). The extracellular matrix makes up the primary portion of tumor microenvironment and is essential for the production of secretory proteins and other components of the outer membrane that affect various intracellular biological processes. On the other hand, down-regulated genes were mainly associated with epithelial cell differentiation, cell cycle, and cell adhesion.

**Table 2:** Number of genes involved in biological process in bladder TICs

Gene ontology	Number of genes	*p* value
Up-regulated genes^*^		
Macromolecular complex assembly	13	0.0036
Response to toxin	4	0.0103
Regulation of organelle organization	6	0.0224
Nucleosome assembly	4	0.0243
Protein-DNA complex assembly	4	0.0298
Down-regulated genes^*^		
Epithelial cell differentiation	9	0.0002
Response to organic substance	17	0.0166
Very-long-chain fatty acid metabolic process	3	0.0225
Cell cycle process	14	0.0228
Cell adhesion	16	0.0265

^*^Only categories with three or more candidate genes are shown.

Our data suggested that, in all of four bladder TICs, a total of three key TFs and 28 differentially expressed genes (including 10 up-regulated and 18 down-regulated genes) constituted a TF-gene regulatory network, which might be the central pathway involved in the regulation of bladder TICs proliferation and survival. The three key TFs were mainly located in the nucleus and correlated with a series of biological process, such as cell proliferation, cell cycle, and regulation of apoptotic process. ETS1, a member of the ETS transcription factor family, has been implicated in tumor angiogenesis and vascularization as a positive response to the hypoxic microenvironment caused by inflammation in tumor tissues [20]. Moreover, ETS1 was shown to contribute to tumor proliferation and invasion by acting in both fibroblasts and neoplastic cells in tumor stroma [21]. The significant (>3-fold) up-regulation of ETS1 might represent an essential step in the development of stem-like characteristics in bladder TICs. EGR1 has been described as a tumor suppressor gene in most human cancers, including fibrosarcoma, glioblastoma, and breast cancer [22-24]. p53 has been shown to be essential for EGR1 role in effecting senescence by activating the p53-MDM2-p19ARF pathway [25, 26]. Therefore, down-regulation of EGR1 might protect tumor cells from apoptosis through inhibition of p53, contributing to their stem-like characteristics. MYC is an oncogenic transcription factor frequently dysregulated in human cancers and is involved in many pathways that support cell oncogenic properties [27, 28]. Although hyperactive MYC would induce uncontrollable cell proliferation and tumor formation, MYC hyperactivation has been shown to also impose a stress on proper mitotic progression [29]. Two recent studies demonstrated that MYC played different roles in tumor and normal tissues. The recently proposed “Amplifier Model” suggests that, instead of affecting specific target genes, MYC both positively and negatively affects the transcription of discrete gene sets and regulates a series of transcription factors, causing unbalance of cellular metabolism and protein synthesis processes and leading to cell carcinogenesis [30, 31].

## MATERIALS AND METHODS

### Cell lines and tissue specimens

Bladder cancer cell lines (T24, 5637, RT4, SW780, UMUC3, TCCSUP, and HT1376) were obtained from the American Type Culture Collection (ATCC, Manassas, VA, USA) and maintained in monolayer cultures in RPMI-1640, DMEM, and MEM medium with 10% fetal bovine serum (Mediatech, Herndon, VA, USA), at 37°C in a 5% CO_2_ humidified atmosphere.

A total of 24 bladder cancer patients were recruited for cancer and distant normal tissue collection at the Department of Urology, The Second People’s Hospital of Shenzhen, Shenzhen, China (Supplementary Table 5). Each patient was required to sign a written informed consent form. Data from patients were analyzed anonymously. All tissue samples were obtained from the surgery room and snap-frozen and stored in liquid nitrogen within 10 min of the resection. TNM staging and histological classification were performed according to World Health Organization (WHO) criteria.

### Cells sorting

Cultured cells were labeled with phycoerythrin-conjugated anti-CD44 antibody (BD PharMingen catalog number: 550989). Flow cytometry analysis and cell isolation were performed on a BD FACSAria II Cell Sorter (Becton Dickinson Immunocytometry Systems, San Jose, CA, USA). Sorted cell suspensions were treated with RNA™safer RNA stabilization reagent (SABiosciences, Frederick, MA, USA) to ensure RNA stability, and stored at -80°C for subsequent RNA isolation.

### Tumor sphere formation assay

Sorted cells were placed in Low Attachment 6-well plates (Corning Incorporated Life Sciences) and cultured in Dulbecco’s modified Eagle’s medium/F12 (Invitrogen) supplemented with 20 ng/ml epidermal growth factor (Sigma), 20 ng/ml basic fibroblast growth factor (Sigma), B27 supplement (Invitrogen), N-2 supplement (Invitrogen), and 1% methylcellulose (Sigma). Spheres were counted under a stereomicroscope after 2 weeks.

### *In vivo* xenograft models

CD44^high^ (TICs) and CD44^low^ (non-TICs) were separately suspended in Matrigel matrix (Becton Dickinson catalog number: 354248), mixed with media at a 1:1 ratio, and injected intradermally into the dorsal skin of NOD/SCID mice using 31-gauge insulin syringes (Becton Dickinson).

### RNA isolation, microarray hybridization, and signal scanning

Cell RNA was extracted using Trizol (Invitrogen, CA, USA) and purified with the RNeasy Mini kit (Qiagen, Düsseldorf, Germany), according to the manufacturer’s instructions. Previous to microarray hybridization, RNA concentration and purity were determined using a UV2800 ultraviolet spectrophotometer (UNIC, New York, NY, USA). RNA concentrations ranged between 100 ng/ml and 1 mg/ml. A260/A280 ratio values ranged between 1.8 and 2.0.

The Affymetrix PrimeView™ Human Gene Expression Array (Affymetrix, Santa Clara, CA, USA) was used to profile differentially expressed genes in bladder TICs *vs*. bladder cancer cells following the manufacturer’s instructions. Briefly, extracted RNA template (1 mg) was reversely transcribed into cDNA and digested into fragments with endonucleases. These fragments were labeled with DNA labeling reagent and labeled cDNAs were hybridized to the microarray via incubation at 45°C and rotated at 60 rpm for 17 h. Following washing and staining, the arrays were scanned using a GeneChip Scanner3000 with GeneChip Operating Software.

### Construction of the transcription factor-gene regulatory network

The TF-gene regulatory network was built by combining information from the bladder cancer TICs gene expression profile (this study) and data from TRED using Cytoscape software version 3.1.1, according to differential gene expression levels and regulatory interactions. The adjacency matrix was constructed by the attribute relationships among all genes and the TFs.

### Real-time quantitative RT-PCR

For real-time qRT-PCR analyses, less than 5 mg total RNA was reverse-transcribed into cDNA using the 1st strand cDNA synthesis kit (Takara, Dalian, China). mRNA expression for human *ETS1*, *EGR1*, *MYC*, *BAX*, *SLC39A6*, and *ITGB1* genes was examined by qRT-PCR using SYBR Premix Ex Taq (Takara) and a Biosystems 7300 Fast Real-Time PCR System. Relative mRNA expression was normalized to *β-actin* mRNA levels using the comparative Ct method (2^-ΔΔCt^, ΔCt = Ct_target_ - Ct_β-actin_, ΔΔCt = ΔCt_CD44_^high^ - ΔCt_CD44_^low^). Primers were designed using the Primer Premier 6 software. Primer sequences are listed in Supplementary Table 6. qRT-PCR data were analyzed using GraphPad Prism Version 5.0; differences between groups in each sample were statistically evaluated by one-tailed Student’s t-test. *P-values* ≤0.05 were considered statistically significant.

## SUPPLEMENTARY MATERIALS FIGURE AND TABLES




