# Supplementary material for: Gene expression profiling and construction of a putative gene regulatory network of bladder cancer tumor-initiating cells
Source: Oncotarget. 2017 Nov 30;8(67):111271–80. doi: 10.18632/oncotarget.22771 (PMC5762320; doi:10.18632/oncotarget.22771)
Supplement: Supplementary file 1 [file oncotarget-08-111271-s001.pdf]

Gene expression profiling and construction of a putative gene regulatory network of bladder cancer tumor-initiating cells

SUPPLEMENTARY MATERIALS

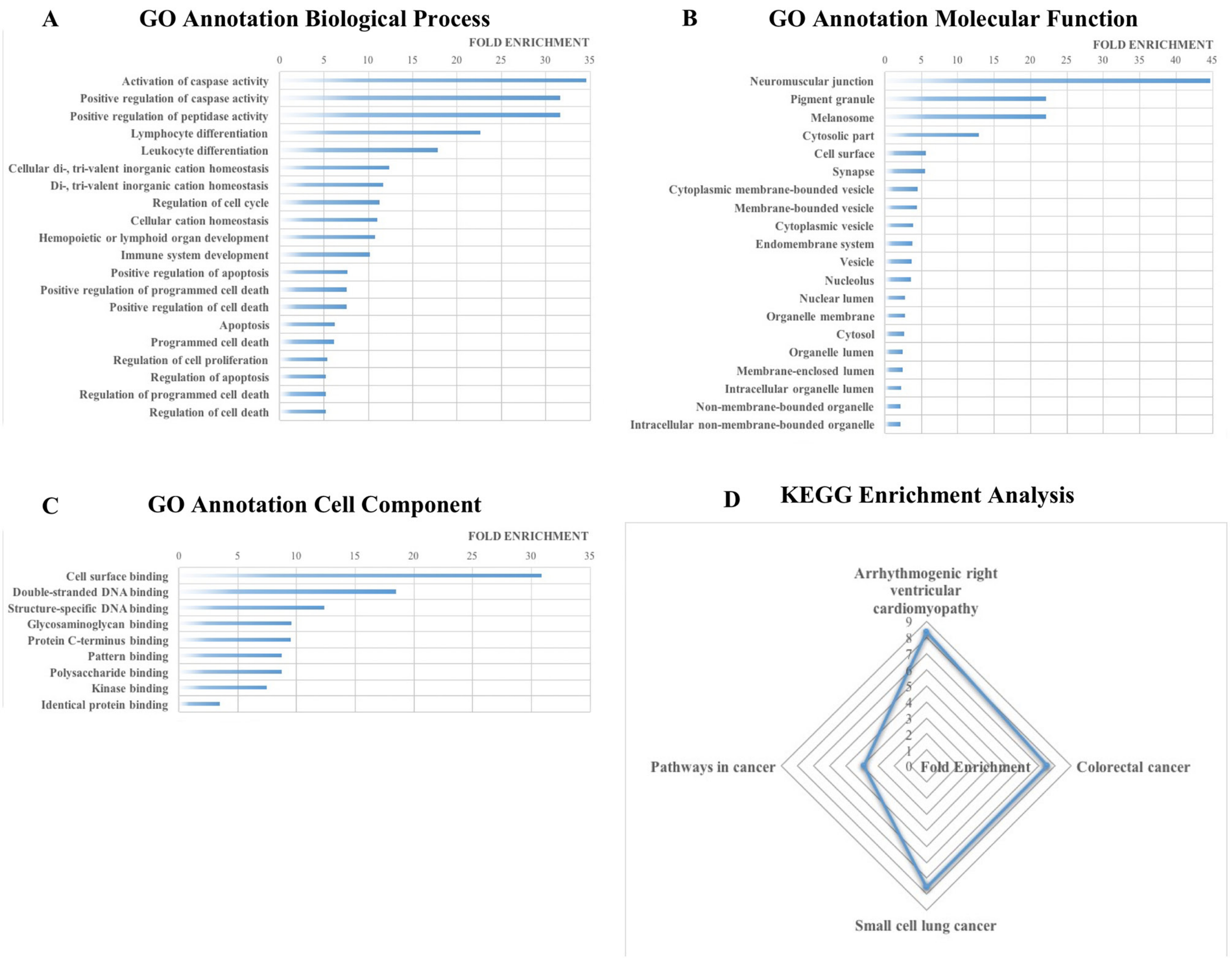

Supplementary Figure 1: GO annotation and KEGG enrichment analysis. **(A-C)** The enriched GO terms for the genes in network. **(D)** The KEGG enriched terms for the genes in network.

Supplementary Table 1: Bladder cancer cell line information

Name	Morphology	Culture properties	Disease	Age	Gender
T24	Epithelial	Adherent	Transitional cell carcinoma	81	Female
5637	Epithelial	Adherent	Grade II carcinoma	68	Male
UM-UC-3	Epithelial	Adherent	Transitional cell carcinoma	——*	Male
RT4	Epithelial	Adherent	Transitional cell papilloma	63	Male
SW780	Epithelial	Adherent	Transitional cell carcinoma	80	Female
TCCSUP	Epithelial	Adherent	Grade IV transitional cell carcinoma	67	Female
HT-1376	Epithelial	Adherent	Grade III carcinoma	58	Female

*Symbol "——" represent the lost information.
The information of each cell from website of American Type Culture Collection, America.

Supplementary Table 2: The differentially expressed genes in CD44high bladder cancer cells among SW780, HT-1376, T24 and 5637 cell lines.

See Supplementary File 1

Supplementary Table 3: The co-differentially expressed genes among different cell lines

	**SW780 vs HT-1376**	**NO.=2**
	**Gene Symbol**	**EntrezID**
1	CARM1	10498
2	ERBB2IP	55914
	**SW780 vs 5637**	**NO.=1**
	**Gene Symbol**	**EntrezID**
1	ATF3	467
	**HT-1376 vs 5637**	**NO.=1**
	**Gene Symbol**	**EntrezID**
1	TNFAIP2	7127
	**T24 vs 5637**	**NO.=4**
	**Gene Symbol**	**EntrezID**
1	MAGOH	4116
2	HIST2H2AC	8338
3	ASS1	445
4	PTGS2	5743

Supplementary Table 4: Information for the dfferentially expressed genes

Cell lines	Genes	EntrezID	Fold	Style	Type
T24	ETS1	2113	3.14156409	TF	UP
5637	EGR1	1958	0.22264572	TF	DOWN
WU	MYC	4609	0.43029815	TF	DOWN
5637	TFRC	7037	2.28993006	Target	UP
5637	RPS25	6230	2.25902629	Target	UP
5637	NDUFB3	4709	2.31100432	Target	UP
5637	PDGFC	56034	2.13165001	Target	UP
5637/T24	MAGOH	4116	4.00811560079473/2.68682770724806	Target	UP
EJ	SLIT1	6585	3.13805512	Target	UP
EJ	LTB	4050	3.13188835	Target	UP
T24	BAX	581	2.11613669	Target	UP
T24	DAD1	1603	2.00420734	Target	UP
WU	LCK	3932	2.1715318	Target	UP
5637	HIST2H2BE	8349	0.43107065	Target	DOWN
5637	CDKN2B	1030	0.48805766	Target	DOWN
EJ	STOM	2040	0.35516003	Target	DOWN
T24	APP	351	0.41488948	Target	DOWN
T24	RPS6	6194	0.27638534	Target	DOWN
T24	MAGEA3	4102	0.28537588	Target	DOWN
T24	ATP2A2	488	0.47729857	Target	DOWN
WU	SLC39A6	25800	0.48736234	Target	DOWN
WU	HMMR	3161	0.40321288	Target	DOWN
WU	ATP6V1G2	534	0.35883122	Target	DOWN
WU	ITGB1	3688	0.47806142	Target	DOWN
WU	UCHL1	7345	0.49969953	Target	DOWN
WU	RECQL	5965	0.40454016	Target	DOWN
WU	ZNF146	7705	0.44076162	Target	DOWN
WU	HPGD	3248	0.48996665	Target	DOWN
WU	F3	2152	0.47588004	Target	DOWN
WU	CTNNB1	1499	0.42167726	Target	DOWN
WU	GLYAT	10249	0.41142418	Target	DOWN

Supplementary Table 5: Clinical information for patients

Clinicopathological details of patients (N=24)	Frequency
**Mean Age (Range)**	59(46-72)
**Gender**	
Male	19
Female	5
**Operation**	
TURBT	19
Partial Bladder Resection	2
Pelvic Tumor Resection	2
Bladder Resection	1
**Histopathological Types**	
High or Moderate	16
Low or Undifferentiated	8
**Clinical Stages**	
T1	18
T2	2
T3	1
T4	2
Ta	1
**Muscular Invasion**	
Yes	5
No	19
**Lymphatic Metastasis**	
Yes	5
No	19
**Mean Tumor Size (cm)**	
<1	19
>1 but <2	3
>5	2

Supplementary Table 6: The qRT-PCR primer sequences

**Primers for specific genes**		
Human beta;*actin*	Forward:	5'- GTCACCAACTGGGACGACAT-3'
	Reverse:	5'- AGGGATAGCACAGCCTGGAT-3'
Mouse beta;*actin*	Forward:	5'- TACCACCATGTACCCAGGCA-3'
	Reverse:	5'- GGAGGAGCAATGATCTTGAT-3'
Human *EST1*	Forward:	5'- GATAGTTGTGATCGCCTCACC -3'
	Reverse:	5'- GTCCTCTGAGTCGAAGCTGTC -3'
Human *Myc*	Forward:	5'- GGCTCCTGGCAAAAGGTCA -3'
	Reverse:	5'- CTGCGTAGTTGTGCTGATGT -3'
Human *EGR1*	Forward:	5'- GGTCAGTGGCCTAGTGAGC-3'
	Reverse:	5'- GTGCCGCTGAGTAAATGGGA-3'
Human *BAX*	Forward:	5'- CCCGAGAGGTCTTTTTCCGAG -3'
	Reverse:	5'- CCAGCCCATGATGGTTCTGAT -3'
Human *SLC39A6*	Forward:	5'- ATGCAAGTCACCACCATAGTCA-3'
	Reverse:	5'- ACGTGGAATCAAAATAGGCACT-3'
Human *ITGB1*	Forward:	5'- CCTACTTCTGCACGATGTGATG-3'
	Reverse:	5'- CCTTTGCTACGGTTGGTTACATT-3'
